# Exploring the Availability of Emergency Contraception in New Brunswick Pharmacies: A Mystery-Client Telephone Study

**DOI:** 10.3390/pharmacy8020076

**Published:** 2020-04-30

**Authors:** Madison Borsella, Angel M. Foster

**Affiliations:** Faculty of Health Sciences, University of Ottawa, Ottawa, ON K1N 6N5, Canada; mbors085@uottawa.ca

**Keywords:** Canada, emergency contraception, mystery-client, New Brunswick

## Abstract

Although levonorgestrel-only emergency contraceptive pills (LNg-ECPs) have been available over the counter in Canada for more than a decade, barriers to access persist. We aimed to obtain information about the availability and cost of LNg-ECPs in New Brunswick. Using a mystery-client study design, we called all 207 non-specialty pharmacies in the province posing as a 17-year-old woman seeking something to prevent pregnancy after sex. We evaluated the information provided for accuracy and quality. The overwhelming majority of pharmacies (n = 180, 87%) had at least one brand of LNg-ECPs in stock; the price averaged CAD28.69 (USD21.65). Although the majority of pharmacy representatives provided accurate information about LNg-ECPs, a small number made incorrect statements about the timeframe for use, side effects, and mechanism of action. In nine interactions (4%) pharmacy representatives incorrectly indicated that a male partner could not obtain LNg-ECPs; none indicated that parental involvement was required to procure LNg-ECPs. None of the pharmacy representatives referenced any other modality of emergency contraception, including ulipristal acetate. Our findings suggest that LNg-ECPs are widely available and that most pharmacy representatives are providing accurate medical and regulatory information. However, supporting the continuing education of pharmacists and pharmacy staff, particularly around alternative modalities of emergency contraception, appears warranted.

## 1. Introduction

Emergency contraceptives are medications or devices that are used after sex to reduce the risk of pregnancy and represent an important but underutilized contraceptive option for women. Globally there are five modalities of emergency contraception (EC) available: the copper-T intrauterine device (IUD), ulipristal acetate (UPA), levonorgestrel-only emergency contraceptive pills (LNg-ECPs), the post-coital use of combined hormonal oral contraceptive pills (the Yuzpe method), and low-dose mifepristone [1]. Except for low-dose mifepristone, all EC modalities are available in Canada. 

New Brunswick is one of the four provinces that make up Canada’s east coast. As the second most populous of Canada’s Atlantic provinces, New Brunswick has a population of 751,171, with almost half living in rural areas [2,3]. Provincial legislation delegates regulatory power to the New Brunswick College of Pharmacists, thus scheduling amendments made by the National Drug Scheduling Advisory Committee are effective immediately [4]. Therefore, the changes made to the status of LNg-ECPs and UPA were adopted in New Brunswick immediately. As a result, LNg-ECPs are approved for over-the-counter sale and UPA is approved for post-coital use as a prescription drug. However, a body of research in Canada has demonstrated that LNg-ECPs are often held behind-the-counter, thereby reducing timely access [5,6]. Little has been written about EC in New Brunswick. However, a national study found that women living in Atlantic Canada generally had lower levels of familiarity with contraceptive methods other than oral contraceptive pills and condoms when compared to their counterparts in other regions [7]. Research in North America also suggests that women often have inadequate information about the effectiveness, timeframe for use, and availability of different modalities of EC, the difference between medication abortion and LNg-ECPs, and effects of LNg-ECPs on fertility and pre-existing pregnancy [8]. But in order to be able to use methods of EC, women must be aware of their existence and have adequate knowledge about how to use them. Health care providers play a critical role in providing evidence-based information and services and combatting misinformation.

We undertook a mystery-client telephone study to explore the availability of LNg-ECPs in New Brunswick pharmacies. Through engaging with all of the non-specialty pharmacies listed on the New Brunswick Pharmacists’ Association website [9], we aimed to obtain information about availability, cost, parental involvement, and male procurement of LNg-ECPs, and identify avenues by which pharmacy provision of EC could be improved. 

## 2. Materials and Methods 

Mystery-client studies conducted in other settings informed our study design [10,11]. Over a 3-week period in December 2018, we called 207 non-specialty pharmacies in order to obtain information about EC.

### 2.1. Data Collection

MB, a New Brunswick native completing her MSc degree in Interdisciplinary Health Sciences at the University of Ottawa (Canada), made all the telephone calls. Using a client profile (see Section 2.2), MB began each interaction with, “Do you have something I could use to prevent pregnancy after sex?” The interactions then unfolded organically, and MB tried to solicit information about availability, cost, parental involvement, and boyfriend procurement. After calling the general phone number and asking to speak with a “pharmacist,” the interactions lasted between 1 and 8 min. 

### 2.2. Client Profile

We created a client profile in order to mimic an authentic encounter and consistently respond to questions during the interaction. Emily is a 17-year-old young woman from an area near the pharmacy seeking something to prevent pregnancy after sex. The night before (approximately 10–20 h earlier depending on the time of the call) she had penile–vaginal intercourse with her boyfriend of 3 months. The condom “broke” during that interaction, which involved ejaculation. Emily is not using any other type of contraception. She does not know how her health insurance works but has insurance “through her parents.” Her period ended about one week prior to the interaction (making her roughly 12 days from the first day of her last menstrual period (LMP)). Emily has heard about something that can prevent pregnancy after sex but does not know the details or the name of the product. If told “Plan B” she can indicate that she has heard that name before. Emily has no health problems, takes no medications, and has never been pregnant. She is open to learning about the copper-T IUD and/or UPA if the pharmacy representative provides information. She has heard of an IUD but does not know that it can be used as EC. Emily has not heard of UPA. We chose this profile to reflect “typical” characteristics of adolescent sexual health knowledge and behaviors, to indicate that the client was likely at risk of unintended pregnancy based on LMP, and to create organic opportunities to explore pharmacy representatives’ knowledge about regulatory issues such as parental consent or boyfriend procurement.

### 2.3. Data Analysis

MB audio-recorded the telephone conversations and then inputted the information into SurveyMonkey^®^ for later export into Microsoft Excel^®^. We used descriptive statistics to classify the pharmacies and provide basic information about the interactions. We analyzed the summaries for content and themes using both deductive and inductive analytic techniques. Regular meetings between MB and her supervisor (AMF) guided our interpretation and we resolved differences through discussion. We did not collect personally identifying information about individual pharmacy representatives and in this article, we have masked all identifying information about pharmacies. We present our results by domains of inquiry.

### 2.4. Ethical Considerations

Based on the criteria laid forth in Article 2.1 of the Tri-Council Policy Statement 2nd Edition [12], the Office of Research Ethics and Integrity at the University of Ottawa determined that this study did not involve “human participants” and therefore did not require Research Ethics Board review.

## 3. Results

### 3.1. Pharmacy Characteristics

We interacted with a representative from all 207 non-specialty pharmacies listed in the New Brunswick Pharmacists’ Association directory and contacted pharmacies in all 15 counties in the province. We present general characteristics of these pharmacies on Table 1. Using Statistics Canada’s population dwelling classifications [13], 65 of the pharmacies were located in Medium Population Centers (MPCs), 125 pharmacies were located in Small Population Centers (SPCs), and 17 were located in rural areas. The majority of the pharmacies were chain stores (n = 159, 77%), 43 (21%) were banner (independently owned but working with a group for marketing and procurement purposes), and 5 (2%) were independent. In some cases, the pharmacy representative identified her/his/their professional position; we include this information on Table 1. The overwhelming majority of pharmacy representatives that we spoke with appeared to be women (n = 165, 80%) based on name and voice.

### 3.2. Availability and Cost of LNg-ECPs

The overwhelming majority of the pharmacies we contacted (n = 180, 87%) had at least one dedicated LNg-ECP product in stock at the time of our call. Of the 26 pharmacies that did not have LNg-ECPs in stock, 12 (46%) gave our client a referral to another pharmacy. Rural pharmacies were more likely than pharmacies in MPCs and SPCs to not stock LNg-ECPs (24% versus 9% and 13%). Pharmacy representatives provided information about the price of LNg-ECPs in 86% of our interactions (n = 178); the average price (all brands) was CAD28.69 (USD21.65). None of the pharmacy representatives we spoke with provided information about UPA or the copper-T IUD.

### 3.3. Accuracy of Medical Information

Although the majority of the pharmacies provided accurate information about LNg-ECPs, some did provide information that was medically inaccurate (n = 26, 13%); we based our assessment of medical accuracy on international guidelines issued by normative bodies [14,15]. Incorrect medical information included the timeframe for use, side effects, and/or the mechanism of action of LNg-ECPs. The most common error involved the timeframe for use (n = 20, 40%); generally, pharmacy representatives stated that LNg-ECPs had to be used less than 72 h after intercourse. As a provider in an SPC stated, “Is it past 24 h? Because I think [LNg-EC] is supposed to be [used] before.”

Thirty-one pharmacy representatives (15%) offered some information about side effects; overwhelmingly they correctly informed the mystery client that these were minor and transient. However, in two cases pharmacy representatives provided confusing and medically inaccurate information about vomiting and when a person should take a second/additional dose of LNg-EC. Two incorrectly stated that estrogen was the active ingredient of “Plan B” and described side effects and risks that were consistent with the use of combined hormonal oral contraceptive pills, patches, or rings. Finally, 15 pharmacy representatives (7%) provided information about mechanism of action of LNg-ECPs with varying degrees of accuracy and precision. 

### 3.4. Accuracy of Regulatory Information

Pharmacy representatives in New Brunswick discussed a number of EC-related service delivery practices that are in conflict with federal regulations, provincial regulations, and best practices. Although we did not specifically ask about the de facto regulatory status of LNg-ECPs, 48 pharmacy representatives (23%) indicated that the pills were not available “on the shelf”. One pharmacy representative in a SPC incorrectly stated, “We keep it back in the pharmacy, and legally, that is where they have to be.” 

In terms of procurement of LNg-ECPs, 9 pharmacy representatives (4%) incorrectly indicated that a male partner could *not* procure the medication. As LNg-ECPs are supposed to be available over the counter, there should be no age, sex, gender, or other restrictions to procurement. However, as one provider in a SPC explained, “You have to have a consultation to decide if you are eligible for it, so we would need to see you.”

Pharmacy representatives were unanimous that a 17-year-old woman could obtain LNg-ECPs without parental consent. However, several pharmacy representatives expressed that younger teens would need to involve their parents. As one provider in a SPC stated with respect to parental involvement, “No, not at 17, [but] 16 would be another story.” 

Finally, four pharmacy representatives (2%) refused to provide any information about EC over the phone. Instead, they indicated that the pharmacist would have to speak to the client in person before they could provide any information about ECPs. 

### 3.5. Personal Questions Asked

Fifty-three pharmacy representatives (26%) asked our mystery client personal questions. These questions pertained to the use of ongoing contraceptive methods (n = 21), general health/sexual health history (n = 14), name/boyfriend’s name (n = 9), weight (n = 5), circumstances behind the need for EC (n = 2), current pregnancy status (n = 2), date of the last menstrual period (n = 2), and other (n = 8). Table 2 summarizes the type of personal questions asked by the pharmacy representatives. 

### 3.6. Quality of the Interactions

**Figure 1:**
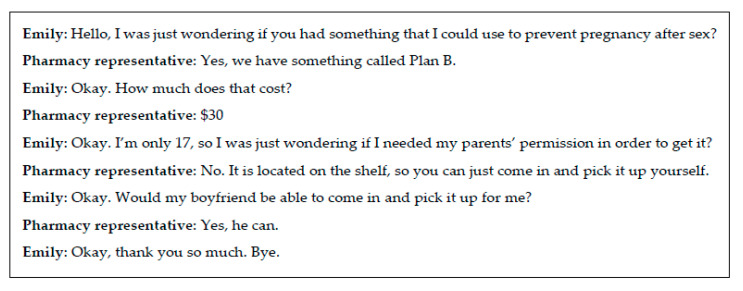
Typical interaction between the mystery client (Emily) and a pharmacy representative.

In terms of quality of interaction, the results were quite variable. The majority of the pharmacy representative (n = 135, 56%) simply answered our questions and did not provide additional information or ask personal questions. We provide an example of this type of interaction as Figure 1. However, in 29 instances (14%) the interaction was both judgmental and uncomfortable. For example, a pharmacist in a SPC said this in an abrasive tone in response to the client stating that she does not use oral contraceptive pills:

So, [oral contraceptive pills] might…be something you want to consider…I just say this because I’ve seen sometimes that people might want to try something like this [EC] on a regular basis, but it is kind of hard on the system, it would not be the most recommended route.

In contrast, 14 pharmacy representatives (7%) went into detail about the location of LNg-ECPs in the pharmacy in order to support anonymity. For example, a pharmacist in a SPC explained:

They are in the section where you get your Canesten, near the check-in counter at the pharmacy and it is called Plan B. It is in a blue box. We can help you find it but if you want to be anonymous, that is why I’m explaining it, so it is aisle number 6, close to the back of the store.

## 4. Discussion

In 2008, Dunn and colleagues found that 87% of Ontario pharmacies had LNg-ECPs in stock [16]; a decade later our findings suggest that pharmacy availability in New Brunswick is comparable. However, over half of the pharmacies that provided information about where LNg-ECPs “live”, and 23% of all pharmacies in the study, indicated that the medication is not actually available on the shelf. This too is consistent with studies in other parts of Canada [5,10,16]. Research from throughout North America has shown that keeping LNg-ECPs behind- rather than over-the-counter impedes timely access, creates opportunities for pharmacists to deny services or ask intrusive questions, decreases patient privacy, and increases the cost [5,16,17]. Stocking LNg-ECPs behind-the-counter also creates barriers for individuals who wish to procure the medication on behalf of a friend or partner. We join the chorus of voices from the reproductive health and rights community and call on pharmacies in New Brunswick to follow federal and provincial regulations and make LNg-ECPs available over the counter.

Pharmacy representatives in our study generally provided medically accurate information about LNg-ECPs and recognized that both minors and men could purchase LNg-ECPs. However, there does appear to be some persistent confusion about the timeframe for use. Although guidance offered by the World Health Organization [14] and the International Consortium for Emergency Contraception [15] is clear that LNg-ECPs can be used up to 120 h after unprotected or underprotected sexual intercourse, the labels on LNg-EC products available in Canada still state the timeframe for use is 72 h [18]. This undoubtedly contributes to a lack of clarity around when LNg-ECPs can be used. However, in most cases in our study, pharmacy representatives indicated that the timeframe for use was *less than* 72 h after sex. This misinformation probably stems from the fact that LNg-ECPs are more effective at reducing the risk of pregnancy the sooner they are used [14]. Continuing education efforts directed at pharmacists and pharmacy staff should continue to emphasize the evidence-based timeframe.

In a number of encounters the pharmacy representative provided detailed information to help the client obtain LNg-ECPs anonymously. These findings are heartening. However, we did have a number of encounters that were uncomfortable and/or judgmental, either because of the types of questions being asked or the overall tone of the interaction. Shoveller and colleagues conducted a study in British Columbia and found that participants reported receiving stigmatizing messages from providers when they sought ECPs and worried that health care providers would consider them irresponsible or promiscuous for requesting post-coital contraception [17]. Highlighting professional and positive encounters with pharmacy representatives may help allay these fears as well as showcase a model for others to follow. 

In 2015, Health Canada approved ulipristal acetate for use as an emergency contraceptive under the brand name ellaOne [19]. Studies have shown that UPA is more effective than levonorgestrel-only EC both in general and when used by women who weigh more than 165 pounds [14,15]. The copper-T IUD is the most effective modality of emergency contraception and if left in place confers ongoing contraceptive benefit [20,21]. However, none of the pharmacy representatives in our study mentioned either UPA or the copper-T IUD as an option to our mystery client. As the client only asked for “something” to prevent pregnancy after sex, this is a missed opportunity. A recent study with pharmacy representatives in Ontario suggests that knowledge of modalities of EC other than LNg-ECPs is limited [22]; we suspect this same dynamic is at play in New Brunswick. Supporting efforts to educate pharmacists and pharmacy staff about other modalities of emergency contraception could help increase access to more effective post-coital methods. 

Our study has several limitations. Notably, although New Brunswick is a bilingual province, we conducted all our calls in English. This enhanced the authenticity of the interaction, but it means we are unable to assess how pharmacy representatives would respond to a Francophone caller. Further, we recognize that a single interaction with a representative from a pharmacy may not reflect the practices of others working at the same institution. However, this approach did allow us to obtain a snapshot of EC availability and accessibility. 

## 5. Conclusions

Our findings suggest that LNg-ECPs are widely available in New Brunswick and that most pharmacy representatives are providing accurate medical and regulatory information. However, encouraging all pharmacies to stock LNg-ECPs over the counter could increase timely access to post-coital contraception. Supporting the continuing education of pharmacists and pharmacy staff, particularly around alternative modalities of emergency contraception, also appears warranted. Encouraging the New Brunswick College of Pharmacists to provide information about all EC modalities to their members could advance this effort.

## Figures and Tables

**Table 1:** Characteristics of pharmacies included in the study (N = 207).

**Area Location of Pharmacy**	
Medium Population Centre	65 (31%)
Small Population Centre	125 (60%)
Rural area	17 (8%)
**Type of Pharmacy**	
Chain	159 (77%)
Banner	43 (20%)
Independent	5 (2%)
**Professional Position of Pharmacy Representative**	
Pharmacist assistant/technician	56 (27%)
Pharmacist	69 (33%)
Unknown	82 (39%)

**Table 2:** Description of the personal questions asked by pharmacy representatives (N = 53).

Topic of Question Asked	Number (%)
Use of other contraceptive methods	21 (10%)
General health/sexual health history	14 (7%)
Name/boyfriend’s name	9 (4%)
Weight	5 (2%)
Circumstances behind need for EC	2 (1%)
Pregnancy status	2 (1%)
Last menstrual period	2 (1%)
Other	8 (4%)

## Abbreviations

EC	Emergency contraception
IUD	intrauterine device
LMP	last menstrual period
LNg-ECPs	levonorgestrel-only emergency contraceptive pills
MPCs	Medium Population Centers
SPCs	Small Population Centers
UPA	ulipristal acetate
